# Seropositivity among Blood Samples Drawn from Suspected Dengue Cases at a Tertiary Care Centre of Nepal: A Descriptive Cross-sectional Study

**DOI:** 10.31729/jnma.5253

**Published:** 2022-02-28

**Authors:** Rupesh Kumar Shreewastav, Manoj Kumar Thakur, Arambam Giridhari Singh

**Affiliations:** 1 Department of Biochemistry, Nobel Medical College Teaching Hospital, Biratnagar, Morang, Nepal; 2 Department of Medicine, Nobel Medical College Teaching Hospital, Biratnagar, Morang, Nepal

**Keywords:** *dengue*, *IgG*, *IgM*, *tertiary care*

## Abstract

**Introduction:**

The cases of dengue fever have been reported more frequently in Nepal these days. The aim of this study was to find the prevalence of seropositivity among blood samples drawn from suspected dengue cases at a tertiary care centre of Nepal.

**Methods:**

A descriptive cross-sectional study was conducted at a tertiary care hospital from 1^st^ June 2017 to 31^st^ October 2018 after getting approval from the Institutional Review Committee (Reference number: 23/2016). A total of 537 suspected dengue patients were selected for the study using convenience sampling. These dengue positive sera were assayed for their reactivity with Immunoglobulin M and Immunoglobulin G present in sera and synthetic peptides of dengue virus by enzyme linked immunosorbent assay. Data was entered and analysed in Microsoft Excel 2016. Point estimate at 95% Confidence Interval was calculated along with frequency, percentage, mean and standard deviation.

**Results:**

Among 537 suspected dengue cases, the seropositivity for dengue was found in 124 (23.09%) (19.52-26.65 at 95% Confidence Interval) of the serum.

**Conclusions:**

The present study revealed that dengue was more prevalent in our setting compared to similar studies. All the synthetic peptides showed reactivity with dengue-positive sera with maximum reactivity shown by RR2 peptide. In dengue-positive sera, RR2 peptide of dengue virus identified more Immunoglobulin M than Immunoglobulin G.

## INTRODUCTION

Dengue fever is a viral disease spread by mosquito of Flaviviridae family.^1,2^ Antigenically, four different serotypes of the dengue virus with 65% genomic similarity exist.^3,4^ Commercially available kits use mixes of inactivated viral preparations or recombinant envelope proteins, so detection of anti-dengue antibodies is problematic.^5^

The envelope (E) protein and non-structural protein (NS1) encoded by dengue virus are very important in the pathogenicity of dengue virus infection and are highly conserved in all strains.^6^ Hence, these two proteins were selected to identify epitopes for detecting anti IgM and IgG antibodies. These epitopes were synthesised as linear peptides with the help of DNA star approach.^7^

The aim of this study was to find the prevalence of seropositivity among blood samples drawn from suspected dengue cases.

## METHODS

This was a descriptive cross-sectional study carried out at a tertiary care hospital from 1^st^ June 2017 to 31^st^ October 2018 after getting approval from the Institutional Review Committee (Reference number: 23/2016) of Nobel Medical College and Teaching Hospital (NMCTH). All the patients, who presented to the emergency and medicine departments of NMCTH, suspected with dengue were the study population. Convenience sampling was done. The sample size was calculated by using the formula:

n = Z^2^ × p × q / e^2^

  = (1.96)^2^ × 0.5 × (1-0.5) / 0.05^2^

  = 385

Where,

n = minimum required sample size
Z = 1.96 at 95% Confidence Interval (CI)
p = prevalence taken as 50% for maximum sample size
q = 1-p
e = margin of error, 5%

The sample size calculated was 385. However, we have included 537 patient's samples from Clinical laboratory services, NMCTH. Dengue-positive serum was diagnosed based on the NS1 antigen in an immunochromatographic card test kit. These denguepositive sera were assayed for their reactivity with IgM and IgG present in the sera and synthetic peptides (RR2, RR3, RR4, RR5, RR6, RR8) of dengue virus by ELISA technique.

At PH 9.6, 400ng of peptide was dissolved in 100ml of 0.05M carbonate bicarbonate buffer. At 37°C for 4 hours, the dissolved peptide was coated in the wells of microtiter plates. The wells were rinsed three times with PBS/0.05% Tween 20 (PBS-T) before being blocked overnight at 4°C with 200ml blocking solution (five percent skimmed milk powder in PBS-T). After washing the plates, Chikungunya (CHIKV), DENV, and negative sera (1:100 dilutions) were added to duplicate wells and incubated at 37°C for two hours. Antigen-antibody complexes were identified using HRP-conjugated goat anti human IgM/IgG antibodies (1:2000). The plates were cleaned as before, and colour was developed with a TMB-containing substrate solution. Adding 100 micro litre of 1N sulphuric acid and optically stopping the reaction and optical density (OD) was measured at 492nm. The synthetic peptide RR2 was tested for nondengue viral reactivity using CHIKV sera. The sera that were neither IgM nor IgG DNEV positive were used as a negative control.

To test the diagnostic efficiency of peptide with patient sera, a cutoff value for optical density (OD) was calculated. The cutoff values for peptide were in the range of 0.31-0.34 based on the seroreactivity of 18 healthy human sera tested for IgM. We looked at the seroreactivity of all dengue positive and 20 chikungunya positive patient sera based on the cutoff value for this peptide. The computed cutoff value for IgG was in the range of 0.33-0.38. There was no seroreactivity with any of the peptides in any of the 20 chikungunya positive and healthy sera. When the OD Value of a human blood sample was found to be greater than the cut-off value when tested for IgM and IgG in an ELISA, it was declared reactive with synthetic peptides.

All the peptides were synthesised, purified and authenticated in the All India Institute of Medical Sciences (AIIMS), New Delhi. The Peptide was selected on hydrophilicity, secondary structures, antigenicity index, amphipathicity, using Bcepred/DNA star software for B cell and T cell prediction. These peptides were synthesised by Fluorenylmethoxycarbonyl Chemistry.^7^ The readymade form of these synthetic peptides were gifted to us by AIIMS to carry out the study in Nepal.

The data was entered and analysed in Microsoft Excel 2016. Point estimate at 95% Confidence Interval was calculated along with frequency, percentage, mean and standard deviation.

## RESULTS

Among 537 suspected dengue cases, the seropositivity for dengue was found in 124 (23.09%) (19.52-26.65 at 95% Confidence Interval) of the serum. Out of 124 dengue positive patients, 71 (57.3%) were male and 53 (42.7%) were female. The mean age of dengue positive patients was 41±19.6 year with a range from 5 to 83 years. The maximum number of dengue positive patients was 47 (37.9%) in the 21-40 years age group. Thirty-six (29.03%) of patients were dengue positive in the 41-60 years age group (Table 1).

**Table 1:** Demographic characteristics positive patients (n= 124).

Characteristics	n (%)
**Gender**	
Male	71 (57.3)
Female	53 (42.7)
**Age (years)**	
≤20	18 (14.51)
21-40	47 (37.9)
41-60	36 (29.03)
≥60	23 (18.54)

All peptides responded with dengue sera, according to an analysis of 124 dengue positive sera verified for NS1 using a determined cutoff value. RR2 detected IgM in 100 (80.6%) sera, RR3 in 93 (75%) sera, RR4 in 96 (77.41%) sera, RR5 in 64 (51.6%) sera, RR6 in 86 (69.35%) sera, and RR8 in 90 (72.58%) sera. IgG antibodies against synthetic peptides were found in several of the dengue positive samples. IgG was identified by RR2 in 60 (48.38%), RR3 in 64 (51.6%), RR4 in 57 (45.96%), RR5 in 53 (42.74%), RR6 in 50 (40.32%), and RR8 in 51 (41.12%) sera (Table 2).

**Table 2:** Sero-reactivity of RR2, RR3, RR4, RR5, RR6 and RR8 peptide with dengue positive serum (n= 124).

Peptides	Antibodies	n (%)
RR2	IgM	100 (80.6)
	IgG	60 (48.38)
RR3	IgM	93 (75)
	IgG	64 (51.6)
RR4	IgM	96 (77.41)
	IgG	57 (45.96)
RR5	IgM	64 (51.6)
	IgG	53 (42.74)
RR6	IgM	86 (69.35)
	IgG	50 (40.32)
RR8	IgM	90 (72.58)
	IgG	51 (41.12)

**Figure 1:**
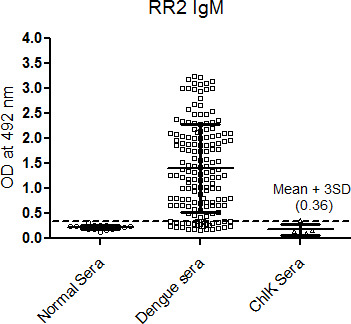
IgM seroreactivity of RR2 peptide with dengue sera prepared by graph pad prism software. (Cut off value represented by a dotted line for RR2 peptide is given as Mean OD±3SD observed with normal sera).

**Figure 2:**
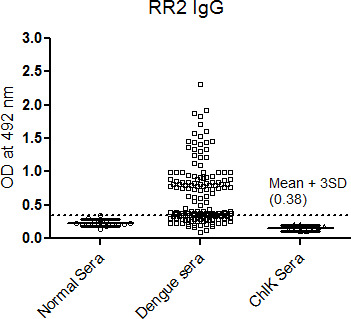
IgG seroreactivity of RR2 peptide with dengue sera prepared by graph pad prism software. (Cut off value represented by a dotted line for RR2 peptide is given as Mean OD±3SD observed with normal sera).

The samples having OD more than the cut-off values were considered reactive. With RR2 peptide the dengue positive sera, the maximum OD obtained was 3.3 and the minimum OD value was 0.5 (Figure 1). Similarly, the IgG dengue positive sera were also reacted with RR2 peptide and found that 60 samples were considered reactive. The maximum OD value obtained was 2.4 and minimum was 0.46 (Figure 2). The data for each sera OD when reacted with other peptides while detecting IgM or IgG are not shown.

## DISCUSSION

Dengue viral infection is a serious health concern. Dengue infection is difficult to control because it necessitates effective vector control as well as early, precise, and strain-specific diagnosis. Dengue fever diagnosis has remained a challenge for disease treatment, particularly in developing countries like Nepal. Therefore, we have made an attempt to study the sero-reactivity of synthetic antigenic peptide of dengue virus with dengue positive sera for the first time in Nepal. This peptide can be used as a tool for the diagnosis of dengue in future. The seropositivity was found in 23.09% of the serum in the present study. Rijal KR, et al. had reported the prevalence of dengue in Nepal in a study, which showed that in 2018, case incidence was about five times higher than in 2016 (incidence rate ratio (IRR): 4.8; 95 % Confidence Interval (CI) 1.5-15.3) and more than 140 times higher in 2019 (IRR: 141.6; 95 % Confidence Interval 45.8-438.4).^8^ A similar finding was reported in a study carried out in Western Uttar Pradesh, India, which revealed that 23% patients were found seropositive either by NS1 Ag or IgM Ab ELISA.^9^ In the present study, among dengue positive patients, 71 (57.3%) were male and 53 (42.7%) were female with mean age of 41 year ranging from five to 83 years. Kumar M, et al. reported that among all dengue seropositive cases in Uttar Pradesh, India , the proportion of male was higher over female with the ratio of (M:F) being 1.54:1 with mean age±standard deviation of all patients was 27.16±14.82 years.^9^ A study conducted in Pakistan had reported that among all dengue positive patients, (72.9%) were males and (27.1%) were females. The patients ranged in age from 6 to 90 years old, with a median age of 30.^10^ We have observed in this study that the maximum number of dengue positive patients was 47 (37.9%) in 21-40 years age group, whereas the study conducted by Raza FA , et al. reported that among dengue positive cases, the maximum 48.5% of dengue positive patients were of 17-30 years old.^10^

In the present study, RR2 peptide detected IgM in 80.6% sera and IgG in 48.38% sera. RR3 detected IgM in 93 (75%) sera, RR4 in 96 (77.41%) sera, RR5 in 64 (51.6%) sera, RR6 in 86 (69.35%) sera, and RR8 in 90 (72.58%) sera. IgG was identified by RR2 in 60 (48.38%), RR3 in 64 (51.6%), RR4 in 57 (45.96%), RR5 in 53 (42.74%), RR6 in 50 (40.32%), and RR8 in 51 (41.12%) sera. In one study, it has been reported that the synthetic peptides detected antibodies in Dengue virus positive serum samples using the predicted epitopes in an indirect ELISA.^11^ In another similar study carried in All India Institute of Medical Sciences, New Delhi demonstrated that synthetic antigenic peptides detected IgM and IgG in Dengue positive sera. Many synthetic peptides were used in combination to form multiple antigenic peptides (1, 2, 3). Multiple antigenic peptide one detected IgM in 96.81% of sera and IgG in 68.15% of sera. Multiple antigenic peptide two discovered IgM positive sera in 94.90% of cases and IgG positive sera in 59.23% of cases. Multiple antigenic peptide three also found IgM and IgG positive sera in 96.17% and 59.87% of cases, respectively.^7^ Mazumdar R, et al. detected dengue specific IgG with some synthetic continuous peptides, spanning over amino acids at positions 51-65, 71-90, 131-170, 196-210, and 246-260 from E protein of Dengue virus-3.^12^ Honda ER, et al. had reported in his study that NS1 specific IgG, IgM and IgA antibodies are found in the sera of patients with dengue fever and dengue hemorrhagic fever.^13^ In many of the studies, these chemically synthesised peptides have been studied as a alternate source of antigen for diagnosis.^14^ RR2 synthetic peptide of dengue virus was reactive with dengue positive sera in the present study, which makes it suitable for the easy and accurate diagnosis of dengue infection in developing country like Nepal, where dengue is endemic. The study also demonstrates that such synthetic peptide can be used easily for dengue diagnosis in the set up of hospitals in countries like Nepal.

There are some limitations of the present study. Dengue-positive patients had been included from one study centre. The results would have been interpreted more reasonably if the more dengue-positive samples would have been enrolled from different epidemic regions of Nepal.

## CONCLUSIONS

The prevalence of dengue was higher in our setting as compared to other similar studies. Among all patients, dengue positivity was found to be more in male than females. The maximum number of patients were reported from the middle younger age group. All the synthetic peptides of dengue virus had shown their reactivity with immunoglobulin M and immunoglobulin G present in the dengue-positive sera. The synthetic peptides detected more IgM than IgG in all dengue-positive sera. Among all the synthetic peptides of dengue virus, RR2 peptide detected more IgM and IgG than other peptides.
